# Cyberbiosecurity: A New Perspective on Protecting U.S. Food and Agricultural System

**DOI:** 10.3389/fbioe.2019.00063

**Published:** 2019-03-29

**Authors:** Susan E. Duncan, Robert Reinhard, Robert C. Williams, Ford Ramsey, Wade Thomason, Kiho Lee, Nancy Dudek, Saied Mostaghimi, Edward Colbert, Randall Murch

**Affiliations:** ^1^Virginia Agricultural Experiment Station, Virginia Tech, Blacksburg, VA, United States; ^2^Department of Food Science and Technology, Virginia Tech, Blacksburg, VA, United States; ^3^Tyson Foods, Chicago, IL, United States; ^4^Department of Agricultural and Applied Economics, Virginia Tech, Blacksburg, VA, United States; ^5^School of Plant and Environmental Sciences, Virginia Tech, Blacksburg, VA, United States; ^6^Department of Animal and Poultry Science, Virginia Tech, Blacksburg, VA, United States; ^7^Biological Systems Engineering, Virginia Tech, Blacksburg, VA, United States; ^8^Hume Center for National Security and Technology, Virginia Tech, Blacksburg, VA, United States; ^9^School of Public and International Affairs, Virginia Tech, Arlington, VA, United States

**Keywords:** plant, animal, food, cyber biosecurity, biosecurity, cyber security, agriculture, bio economy

## Abstract

Our national data and infrastructure security issues affecting the “bioeconomy” are evolving rapidly. Simultaneously, the conversation about cyber security of the U.S. food and agricultural system (cyber biosecurity) is incomplete and disjointed. The food and agricultural production sectors influence over 20% of the nation's economy ($6.7T) and 15% of U.S. employment (43.3M jobs). The food and agricultural sectors are immensely diverse and they require advanced technologies and efficiencies that rely on computer technologies, big data, cloud-based data storage, and internet accessibility. There is a *critical need* to safeguard the cyber biosecurity of our bio economy, but currently protections are minimal and do not broadly exist across the food and agricultural system. Using the food safety management Hazard Analysis Critical Control Point system concept as an introductory point of reference, we identify important features in broad food and agricultural production and food systems: dairy, food animals, row crops, fruits and vegetables, and environmental resources (water). This analysis explores the relevant concepts of cyber biosecurity from food production to the end product user (such as the consumer) and considers the integration of diverse transportation, supplier, and retailer networks. We describe common challenges and unique barriers across these systems and recommend solutions to advance the role of cyber biosecurity in the food and agricultural sectors.

## Introduction: Food and Agricultural Cyberbiosecurity at the Interface of Biosecurity and Cybersecurity

Public trust and confidence in the food supply are critical and influential on acceptance of data-driven innovations and technologies within the food and agriculture systems (Fd+Ag). Cyberbiosecurity is a nascent paradigm and discipline at the interface of biosafety/biosecurity, cyber security, and cyber-physical security (Murch et al., 2018, Figure 1). This new discipline has emerged alongside “big data” with the extensive and ever-increasing reliance of the life sciences on information systems technologies, rapid and profitable expansion of life science discoveries, and the growth of the U.S. bio economy. Protecting biological data and information within the life sciences has unique differences from the more familiar biosafety and biosecurity approaches (Peccoud et al., 2017). While the latter two categories address biological risks and threats, they do not protect against harm created when computational and information technology-dependent systems are threatened or corrupted. Just as food safety regulations target the protection of human health, incorporating cyber biosecurity strategies for the Fd+Ag system is a protective step in securing the food supply. Such efforts have the power to positively influence lives and protect the bio economy. Cyberbiosecurity can improve the security and stability of the domestic and global Fd+Ag system. Innovation in the U.S. Fd+Ag system is routinely studied and adopted around the globe. The U.S. can provide insight and leadership in cyber biosecurity of the global Fd+Ag systems.

**Figure 1:**
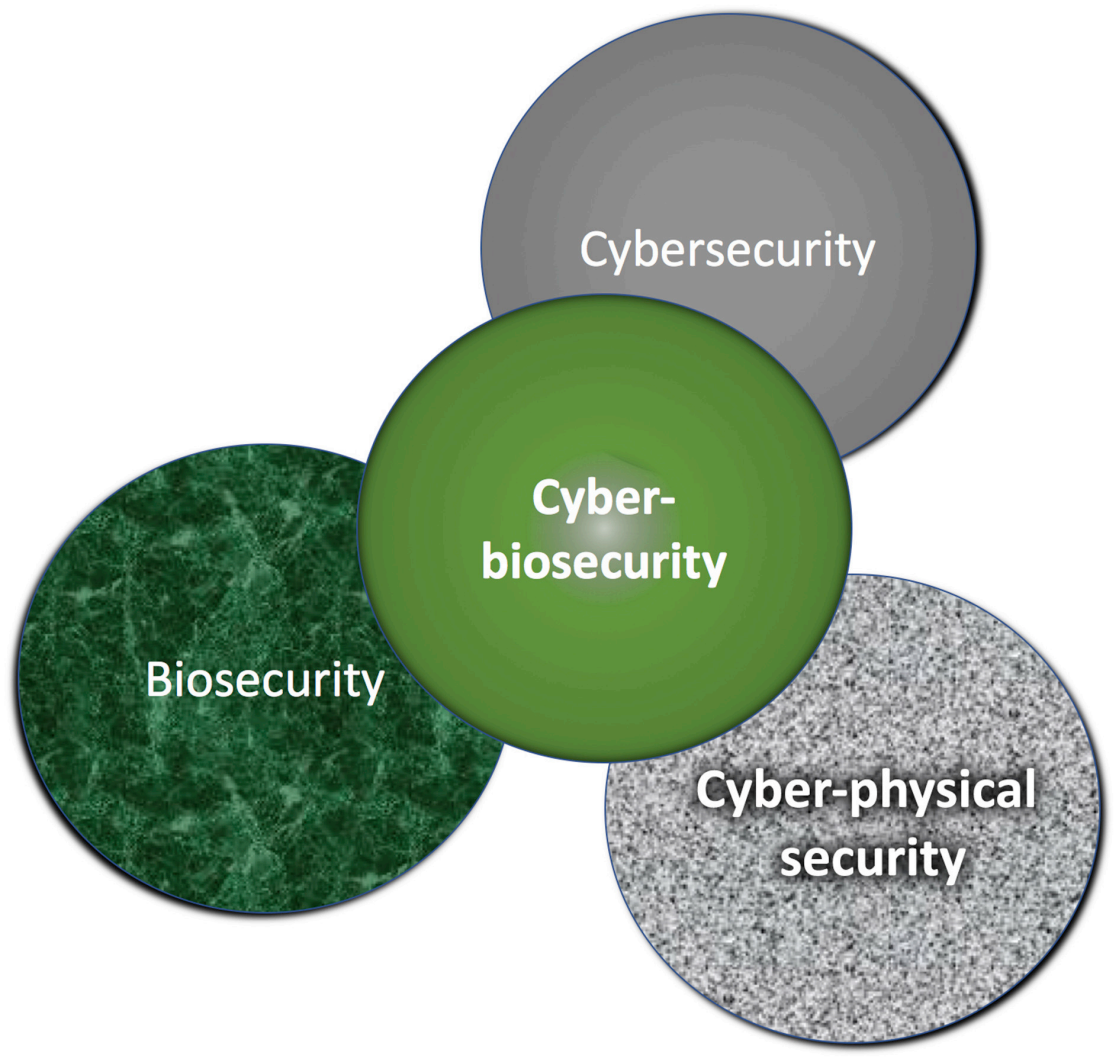
Cyberbiosecurity is an emerging discipline for protecting life sciences data, functions and operations (or infrastructure), and the bio economy.

Integrated scientific, mathematical, computational, and engineering advancements in regenerative biology, genetics and breeding technologies, plant-derived vaccine and animal therapies, biological design and testing automation, and other activities are rapidly leading to development of biotechnological and agricultural applications of direct relevance to the Fd+Ag system (The National Academies of Sciences *Engineering and Medicine (NASEM)*, 2014; Wintle et al., 2017). The translation and application of data-driven technologies for precision agriculture, autonomous systems, bio-automated processing and data recording, and other technologies yields large data sets of economic and bio-based information for agribusinesses (Sykuta, 2016). Such advances require high throughput processing, data management and integration, bio-automation, and other computer-based management of biological data. These advances increase efficiencies, decision processes, and output within the food and agricultural system. However, such information is susceptible to ownership policy challenges, theft, and cyber-attack as users may not be alert to potential vulnerabilities nor be trained in effective protections and security strategies (Sykuta, 2016; Boghossian et al., 2018). Unprotected or weakly protected systems are susceptible to unwanted surveillance, intrusions into data systems, and cyber-activities targeted toward malicious attack. Cyberbiosecurity threats include inappropriate access to systems, data, or analytical technologies and the use or corruption of the information accessed to cause harm within life science-focused research, production, processing, and use. Examples of data-driven, high-value food and agricultural products susceptible to cyber threat include high-yielding and specialty agricultural crops, high performance livestock, biopharma fermented molecules developed through advanced breeding and genomics, biotechnology advancements, and “big data” analyses (The National Academies of Sciences Engineering and Medicine, 2015). As technology advances, all parts of society, from governmental agencies to public health and manufacturing, rely more on advanced biological systems with big data and technologies that utilize such information. The identification and mitigation of cyber biosecurity threats will become increasingly important.

## Vulnerability of the Food and Agricultural System and the Bioeconomy

The U.S. Fd+Ag system, influencing 20% ($6.7T) of the domestic bio economy (Feeding the Economy, 2018), represents a significant risk to global food security. The data science market value for agriculture is estimated in excess of $20B (Sykuta, 2016). The Fd+Ag system is composed of many sectors that are not well-integrated, is widely dispersed geographically, and has huge diversity in size (number of employees) and capacity. Most of the economic value in the Fd+Ag system is generated by large, multinational corporate enterprises. Conversely, small family-owned farming operations account for 90% of U.S. farms, which yield 24% of the value of agricultural production (MacDonald and Hoppe, 2017). The family small-business agricultural enterprise (family farm) has economic and social distinctions from corporate farms. Small farm producers view their data with a sense of personal privacy and protection (Sykuta, 2016). Small businesses often use their internet-linked home computer for both personal and business activities, increasing the risk of cyber-attack (United States Department of Agriculture. National Agricultural Statistics Service., 2013; Geil et al., 2018); over 20% of small businesses get hacked (Geil et al., 2018). Generally, small farms and agribusinesses are not comfortable adopting computer security technology (selecting, configuring, managing) although they recognize its relevance and value. Moderate-sized agribusinesses, including many food processing companies and supporting industries, are vulnerable since cyber-attacks are often targeted against organizations with <100 employees (Geil et al., 2018). The Fd+Ag system includes military food production, such as the manufacturing of packaged meals for soldiers, which has a high potential for sabotage (Colbert et al., 2018). It is important to note that attackers need not know details of the food manufacturing process. Attackers need only know technical methods for exploiting the machinery or the process, such as lowering the temperature on meat cookers before packaging (Colbert et al., 2015a,b).

The incorporation of cyber-based technologies and data driven solutions in farm production, food processing, supplier industries, transport of goods, regulatory oversight, and marketing sales and communication with consumers creates a paradigm shift (Boghossian et al., 2018). Cloud-based storage of large data sets, use of open-sourced or internet/cloud-based software, and corporate management of proprietary software each increase opportunities for data access by unauthorized users. Within the Fd+Ag system, the use of biological and genetic analytical technologies within research laboratories is widespread for the evaluation of food quality, identification of zoonotic disease, and animal and plant health. Additionally, the use of bioinformatics and genetic technologies is enhancing the rate of development of new products and crops. Public trust and acceptance are key to incorporating advanced technologies into the Fd+Ag system (United States Department of Agriculture National Institute for Food and Agriculture, 2016; Wintle et al., 2017). Interdependency of information technology with biological output creates opportunities for new bio-threats, which can harm public trust; transparency is valued (The National Academies of Sciences Engineering and Medicine, 2015). When public opinion is turned against a technical advancement, policy and protection strategies may cause more harm than the actual threat itself (Wintle et al., 2017).

Holistically, the ramifications of a failure to provide cyber biosecurity of the Fd+Ag system fall into several general categories (Boghossian et al., 2018):

- Threats to confidentiality—data privacy
  ◦ Data exposure (e.g., naïve exposure of data by individuals, cyber security gaps in small businesses, or laboratories to potential threats);
  ◦ Capturing private data with intent to aggregate data for profit or predictive advantage.
- Threats to integrity—theft or destruction of intellectual property/productivity disruptions, and safety risks
  ◦ Intellectual property theft (e.g., advances in plant and animal varieties and genetics)
  ◦ Manipulation of critical automated (computer-based) processes (e.g., thermal processing time and temperature for food safety);
  ◦ Seizing control of robotics or autonomous vehicles (e.g., failure to perform, overriding of precise function).
- ∙ Threats to availability—disruption of agricultural/food production and supply.
- Misinformation influencing trust and cooperation within the Fd+Ag system and/or consumers.
- Lack of equipment, supplies, or end-products to meet expectations;
- Lack of ability to perform vulnerability assessments and develop emergency response plans (e.g., protection of rivers, surface waters, and drinking water supplies).

The food and agricultural industries are at a critical point as the development and use of biological, genetic, precision, and information technologies expand and intersect. Collectively, there is a need to evaluate potential liabilities and understand the vulnerabilities of biological and genetic data systems.

## Risk Assessment, Critical Control Points, and Regulatory Options

Cybersecurity risk assessment for industrial control systems (ICS) is advancing rapidly. Cherdantseva et al. (2016) reviewed 24 different cyber security risk assessment methods relevant to ICS. Applications of such risk assessment approaches in Fd+Ag sectors have not been evaluated and the complexity and diversity of the Fd+Ag system may not conform to the current cyber security risk assessment methods. Cyberbiosecurity risk assessment strategies that address the unique security challenges at the intersection of the biological, physical, and cyberspace are important for protecting the Fd+Ag system.

Food manufacturers use the principles of Hazard Analysis and Critical Control Points (HACCP) to assure the production of safe products. HACCP is a familiar risk assessment process within the Fd+Ag system. This management system looks at the likely occurrence of a chemical, biological, or physical food safety hazard in the manufacturing process and the controls that can be put in place to reduce, eliminate, or control the potential hazard. HACCP principles use critical control points (CCPs) as steps in a process where specific controls can be implemented to control, reduce, or eliminate a hazard. HACCP principles are used around the world for the production of safe food products and are required by USDA Food Safety Inspection Service and the U.S. FDA. A risk matrix (Supplemental Material, Table 1) may be used to identify potential vulnerabilities and estimate likelihood of occurrence with the potential public health and financial consequences. An example using HACCP principles for an assessment of an Industrial Laboratory processing biological and genetic materials is presented in the Supplemental Materials. In this specific example, two CCPs (alternative supplier verification of biological and genetic materials program, and cyber biosecurity data verification program) were identified to mitigate potential risks. Four control point programs (supplier approval; employee training; security programs; and good laboratory standard operating procedures) were identified to support the overarching process for cyber biosecurity.

Several economic problems confront policymakers when addressing cyber biosecurity in the Fd+Ag sector. The most pressing concerns are externalities caused by the networked nature of the system and the misaligned incentives of individual agents. The risks associated with cyber biosecurity threats and harm to society are likely to be larger than the losses suffered by an individual entity; individual firms may not have incentives to provide socially optimal levels of security for the network. Furthermore, if agents know that their own protection depends on security investments made by others, they may become free-riders. Again, this results in inadequate private provision of the public good or security of the network (Varian, 2004).

Multiple regulatory and policy options exist to counter threats to the Fd+Ag system. In some cases, it may be easier to implement protections within the Fd+Ag sector because agribusinesses are already subject to relatively strict disclosure regulations. Information disclosure provides regulators with the data necessary to align individual incentives with the security of the system as a whole. This could be done with top-down regulation, changes to the assignment of liability, or the development of market based systems for the control of cyber biosecurity risks. For instance, the development of cyber biosecurity insurance markets could be encouraged. Regardless of eventual policy measures, it will be important to ensure that the costs of protecting the system are properly aligned with the probabilities of loss and magnitudes of loss associated with cyber biosecurity threats. The most efficient methods of securing the Fd+Ag system are likely to rely on a variety of regulatory approaches.

## Considering the Diversity Within and Across Plant, Animal, and Environmental Sectors of the Food and Agricultural System

The HACCP concept assesses risk and establishes CCPs for a specific facility and cannot be generalized effectively to all food manufacturing plants. Applying this concept for cyber biosecurity risk, control points, and CCPs, therefore, is challenged by the diversity of enterprises within a sector and across the Fd+Ag system. Within each sector are unique suppliers providing biological material, chemicals and ingredients, robotics and machinery, software, data, and data storage systems. Some of security measures are encompassed by cyber security, cyberphysical security, and biosecurity/biosafety practices, at least for large corporate entities with sufficient resources. However, an unsecured system from a small agribusiness supplier, producer, processor, or commodity cooperative, could introduce risk.

**Figure 2:**
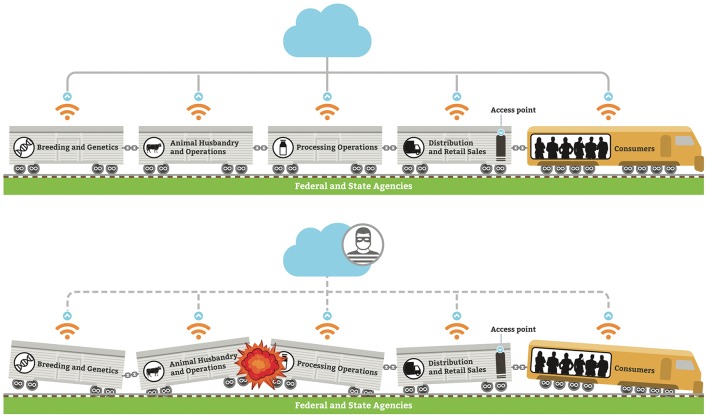
**(Top)** Fd+Ag system for each commodity sector is a sequence of stages, with limited communications and sharing of data between each; **(bottom)** if a cyber-biosecurity event occurs, it can have catastrophic effect on the entire Fd+Ag system.

We use the illustration of a train with multiple boxcars as an example of various sectors within one commodity sector of the Fd+Ag system (Figure 2, top). The various cars represent the transition from genetics and breeding through production, processing, distribution, and consumer purchase/use. The exchange of information between the different sectors is often limited, as illustrated by the couplings. The role of the federal government policies and programs provide support and guidance (tracks). Suppliers and other support systems access one or more sectors within a commodity system. The system is driven (engine) by general public (consumers) acceptance of practices and goods, or their fear and mistrust if a risk or threat is perceived. If any stage “derails” or if any supporting agency or organization “buckles” due to a cyber-biosecurity threat or attack, the entire system is at risk, with subsequent risk to the U.S. food supply and the bio economy (Figure 2, bottom). Currently, the cyber security industry is not visibly involved in protecting biological data interfacing with the cyber-physical infrastructure supporting the Fd+Ag system.

Some potential mitigations to the issues are possible. Cyberbiosecurity planning and implementation are needed to protect the intellectual and physical (data) property associated with such Fd+Ag priorities. Examples include:

- Plant and animal germplasm, such as old world corn germplasm, microbiology collection (pathogens, fermentation, microbiome) repositories, including economic assessment and protection of data sharing;
- Biocontrolled systems or processes, such as “smart” technology greenhouse data;
- Animal and plant disease diagnostic networks and information sharing;
- Fermentation processing and thermal processing control parameters;
- Freshwater and drinking water supplies and treatment systems.

We further illustrate by outlining some unique considerations for various Fd+Ag commodities.

- Dairy: Selection of genetics for breeding is key to the high milk production in the U.S. dairy industry. Genetic data is highly evaluated as part of the process for breeding. Milk production records are important for establishing high performance animals. While there are some very large dairy herds (>2,000 animals), the U.S. dairy industry is dominated by small to medium farms, many of whom sell their milk through a cooperative structure. Herd health records and drug use are regulated. Data security is variable, and often limited. Fluid milk and dairy food processors do not have detailed records of individual cow production or farm production practices, creating a gap in tracing of information and potential for data breach. Processors utilize computer systems for maintaining processing temperatures, ingredient additions, sanitizing, and cleaning steps.
- Food Animals: Selective breeding is critical to maximize genetic gain during food animal production. For instance, multiple line of breeds are incorporated into swine production to enhance heterogeneity. Pedigree information of the breeds significantly influences selection of founders for the production system. Breach or manipulation of the information can lead to a devastating loss to producers. Recent development in genomic-based selection strategies (Sellner et al, 2007) may also be vulnerable to cyber biosecurity threats as the genomic information can be targeted or exploited. Potential application of genome editing technology in food animals (Telugu et al., 2017) may also generate novel genetic information that could dramatically improve productivity of food animals.
- Row Crops: Similar to the dairy industry, the row crop sector consists of a large number of farms of varying size. Grain is typically comingled at the first point of sale and often aggregated further during the process of storage and handling, greatly limiting traceability (Golan et al., 2004). Modern farms using precision agriculture technologies generate enormous amounts of data, about everything from soil conditions to machinery performance and location; such information is often controlled by agriculture technology providers (Sykuta, 2016; Boghossian et al., 2018). Securing data and preventing breaches across all these systems is difficult and is frequently an afterthought by the actual users (Ferris, 2017). Individual producer data is often sent directly to a third party entity for data storage, cleaning, and processing. Many aggregate data and use this as market information or sell it to other companies who do. Commodity traders may use some data streams to guide investment. Anonymization typically occurs at the time of aggregation but questions exist about the effectiveness of these techniques. After transfer, data security becomes the responsibility of the third party data management company, but these entities are themselves not immune from security breaches and would be vulnerable to security issues inserted upstream at the farm or machinery level. Finally, commodity markets are strongly influenced by crop production estimates generated by surveys of farmers and the agriculture industry.
- Fruits and Vegetables: Fresh fruits and vegetables are leading sources for foodborne illness in the United States (Callejón et al., 2015). Furthermore, even in the absence of foodborne illness outbreaks, fresh produce recalls occur regularly due to the presence of potential harmful microorganisms. Fresh produce available for sale in local markets may have been produced in one of many locations throughout the nation or from one of many countries around the world. The production, sorting, grading, commingling, transporting, marketing and sale of fresh fruits and vegetables is complex, and involves numerous industry actors with varying roles. Tracking fresh produce from initial production through consumption is critical to limit the potential for and impact of foodborne illness outbreaks. Accurate product information and rapid access to data is essential to identify contaminated product in the market, prevent or limit foodborne illness, limit the damage to non-implicated producers, and maintain consumer confidence. Access to product tracking and microbiological data is increasing in the fresh produce industry.
- Environmental resources (water): Drinking water safety is extremely important on-farm, for food processing, ensuring the consumers' health and for the proper functioning of the ecosystem. The proportion of the world's population consuming drinking water from certified and controlled water sources is about 90% and still increasing (Vieira, 2011). However, 2.3 billion people worldwide suffer from diseases related to drinking water. Over the past three decades, significant drinking water contamination incidents have occurred in developing as well as developed countries, creating health problems for consumers (Hamilton et al., 2006; Tsoukalas and Tsitsifli, 2018). Traditional risk management systems, based on addressing and correcting the failure after its occurrence, are inadequate to deal with potential cyber biosecurity threats (as the cyber security landscape is changing rapidly as technology continues to advance). Given the severity of risk and potential harm, cyber biosecurity must be given a high priority for the drinking water management and treatment sector (Germano, 2018).

## Conclusions: Moving Toward Solutions

The complex and vastly diverse enterprises within the Fd+Ag system increases vulnerability of our food supply and threatens our ability to contribute to the global food supply. Rapid advancements in technologies and adoption into the Fd+Ag sectors increase the risks for cyber biosecurity threats and attacks. The current Fd+Ag workforce has limited knowledge or training appropriate to evaluate and protect the vast amount of data generated by these technologies. The cyber security industry is not well-prepared to address the unique structure and functions within Fd+Ag system. Protecting the Fd+Ag system includes (1) developing and characterizing effective cyber biosecurity risk assessment and mitigation strategies; (2) developing and preparing the current and future workforce to identify, address and adopt effective cyber biosecurity strategies; (3) considering policy and regulations, including insurance, for protection within and across the Fd+Ag system; and (4) effectively communicating within sector and across the Fd+Ag system (United States Department of Agriculture National Institute for Food and Agriculture, 2016). Awareness, knowledge, adoption, and frequent evaluation of cyber biosecurity plans and strategies among and within all Fd+Ag sectors is essential. A multidisciplinary approach integrating expertise in agriculture, food, engineering, computer science, and cyber security is needed for filling this gap. The USDA, in consultation with academic, public and private sector experts and representation from sectors within the Fd+Ag system, should lead an initiative for developing a planned approach to addressing cyber biosecurity. Private and public funding is needed to support research priorities and implementation strategies. Checkoff funding mechanisms or cooperative agreements, which are common within the Fd+Ag commodity systems, may be options for assisting small to moderate-sized agribusinesses. Workforce development, effective communication strategies, and cooperation across sectors and industries will help increase support and compliance, reducing the risks and providing increased protection for the U.S. bio economy and our domestic and global food supply.

## Author Contributions

SD lead author, responsible for structure, content, and figure; responsible for considering, incorporation co-author contributions and suggested edits; responsible for final version. RR provided draft content related to HACCP and post-harvest processing and cyber biosecurity; contributed, reviewed, and edited the manuscript. RW contributed, reviewed, and edited content related to HACCP and post-harvest processing and biosecurity; reviewed and critiqued manuscript to ensure quality and flow. FR contributed, reviewed, and edited content related to food and agriculture system influence on bio economy; reviewed and critiqued manuscript to ensure quality and flow. WT contributed, reviewed, and edited content related to cyber biosecurity in agriculture (pre-harvest; crop, soil, and environment); reviewed and critiqued manuscript to ensure quality and flow. KL contributed, reviewed, and edited content related to cyber biosecurity in agriculture (pre-harvest; animal breeding and genetics); reviewed and critiqued manuscript to ensure quality and flow. ND contributions to sections relating to biotechnology; overall quality assurance and readability reviews and modifications. SM contributed, reviewed, and edited content related to cyber biosecurity in food and agricultural system and the environment; reviewed and critiqued manuscript to ensure quality and flow. EC contributed, reviewed, and edited content related to cyber security, data sources, and integration into the food and agricultural system; reviewed and critiqued manuscript to ensure quality and flow. RM co-originator of the cyber biosecurity concept; co-originator of the concepts relating to food and agricultural system; contributed, reviewed, and edited content related to cyber biosecurity, data sources, and integration into the food and agricultural system; reviewed and critiqued manuscript to ensure quality, flow, and relevance to the targeted audience.

### Conflict of Interest Statement

RR is employed by Tyson Foods. The remaining authors declare that the research was conducted in the absence of any commercial or financial relationships that could be construed as a potential conflict of interest.
