# Supplementary material for: Cyberbiosecurity: A New Perspective on Protecting U.S. Food and Agricultural System
Source: Front Bioeng Biotechnol. 2019 Mar 29;7:63. doi: 10.3389/fbioe.2019.00063 (PMC6450256; doi:10.3389/fbioe.2019.00063)
Supplement: Supplementary file 1 [file Data_Sheet_1.PDF]

Supplemental Material

Threats associated with cyberbiosecurity can be identified and classified in a risk matrix using what is known from cybersecurity, cyber-physical security and biosecurity. The relative likelihood of an event can be estimated based on other cybersecurity threats and vulnerability, how those threats could be used against biological and genetic targets and the sophistication required to execute an attack. By plotting the risk estimates in a bubble chart, the relationship between likelihood and public health consequences, (y- and x-axis, respectively) and financial impact (size of bubble), the relative risks can be considered (https://www.ucop.edu/enterprise-risk-management/tools-templates/risk-assessment-toolbox-content/risk-ranking-tool.html). Table 1 illustrates an example using biotechnology applications for the Fd+Ag system.

Supplemental Material List of Tables

Table 1. Illustration of cyberbiosecurity threat analysis, including likelihood of threat, public health consequence, and financial impact.

**Risk**	**Likelihood (1=low; 10=high)**	**Public Health Consequence (1=low; 10=high)**	**Financial Consequence (1=low; 10=high)**
Use of a genetic material different than expected	8	2	2
Receipt of a DNA sequence with harmful sequences	2.5	8	8
Corruption of critical biological data	7	8	9
Use of genetic code with steal virus or attributes	1	10	10
Compromise DNA/laboratory software	6.5	3.5	5
Compromise biosecurity systems/access (i.e. employee badges and key code)	7	1	4
Synthetic DNA coded with computer malware	5	2	9
Corruption of genetic stock information	4	4	5
Genetic based (PCR) primer manipulation	3	3	3

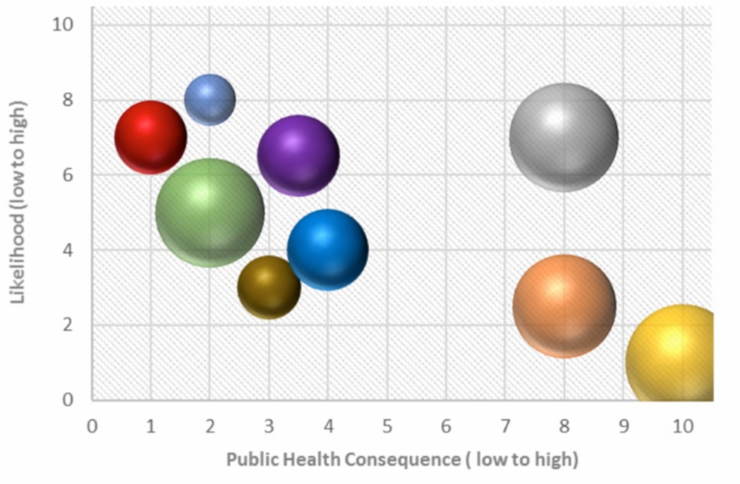

Bubble color corresponds to Risk. Location on vertical axis relates to likelihood of event occurring. Location on horizontal axis reflects potential for a public health consequence. Size of bubble reflects financial impact (larger bubble, greater financial impact).

Data is not validated but presented as an illustration.

Table 2.

Example of Laboratory Cyberbiosecurity When Using Biological and Genetic Material.

**1**<br><br>**Process Step**	**2**<br><br>**Cyberbiosecurity Hazard**	**3**<br><br>**Reasonably Likely to Occur at this Step?**	**4**<br><br>**Basis**	**5**<br>**What measures could be applied to prevent, eliminate, or reduce hazard to an acceptable level**	**6**<br><br>**Critical Control Point?**
**Hazard Analysis**					
Receiving – materials, supplies, biological and genetic samples	Biological or Genetic material<br>• different than expected<br>• receipt of DNA sequence data with harmful sequences<br>• use of genetic code with stealth virus or attributes<br>• synthetic DNA coded with computer malware	Yes	• Genetic material could be incorrectly supplied.	Subsequent process verification steps will identify and control incorrectly supplied material Process verification program for biological and genetic material should be established. Required Program = **Supply Approval Program**	No; control point program
			• Biological or genetic material could be intentionally manipulated or wrong material intentionally supplied (external)	A CCP needs to be in place for validation of biological and genetic materials from alternative suppliers. **Alternative Supplier Validation Program**	**Yes; CCP1**

**Process Steps 1 CPs and CCPs**					
Dilution, pre-enrichment & enrichment	Biological material not controlled in laboratory or manipulated	No	Training and awareness of employees is critical. Process should be designed to assure proper handling of biological and genetic materials when completing laboratory analysis.	**Employee Training**	No
	Corruption of Critical Biological data	No	Proper laboratory practices and purification of samples.	**Laboratory Standard Operating Procedures**	No
	Compromise Biosecurity systems/access (i.e. employee badges and key code)	No	Access is limited and controlled through security programs with proper physical security protocols and system cybersecurity.	**Security Program and Training**	No
	Genetic based (PCR) primer manipulation	No	Good laboratory practices with proper positive and negative controls.	**Good Laboratory Practices Program**	No
**Process Step 2 CPs and CCPs**					
Plating, purifying, & biochemical identification	Biological material not controlled in laboratory or manipulated	No	Training and awareness of employees is critical. Process should be designed to assure proper handling of biological and genetic materials when	**Employee Training**	No

			completing laboratory analysis.		
	Corruption of Critical Biological data	No	Proper laboratory practices and purification of samples.	**Laboratory Standard Operating Procedures**	No
	Compromise Biosecurity systems/access (i.e. employee badges and key code)	No	Access is limited and controlled through security programs with proper physical security protocols and system cybersecurity.	**Security Program and Training**	No
	Genetic based (PCR) primer manipulation	No	Good laboratory practices with proper positive and negative controls.	**Good Laboratory Practices Program**	No
**Process Step 3 CPs and CCPs**					
Genetic Analysis & Metagenomics	Biological material not controlled in laboratory or manipulated	No	Training and awareness of employees is critical. Process should be designed to assure proper handling of biological and genetic materials when completing laboratory analysis.	**Employee Training**	No
	Corruption of Critical Biological data	No	Proper laboratory practices and purification of samples.	**Laboratory Standard Operating Procedures**	No
	Compromise Biosecurity systems/access (i.e.	No	Access is limited and controlled through security programs with proper physical	**Security Program and Training**	No

	employee badges and key code)		security protocols and system cybersecurity.		
	Genetic based (PCR) primer manipulation	No	Good laboratory practices with proper positive and negative controls.	**Good Laboratory Practices Program**	No
**Process Step 4 CPs and CCPs**					
Research data records, storage, taxonomic database review	Compromise DNA/laboratory software	Yes	Since laboratory will be using 3rd party data bases and software, the risk of malware and manipulation exists. Verification of biological and genetic data prior to uploading information will identify changes or potential manipulation of data with malware codes.	Antivirus malware software should be used for all data received from external database and software systems. Data should be verified to assure information was not corrupted or changed when returned from 3rd party software system.<br><br>**Cyberbiosecurity Data Verification Program**	**Yes; CCP2**
	Corruption of genetic stock identification	No	Internal cybersecurity program will prevent malware corrupting software systems. Internal laboratory standard operating procedures will assure appropriate handling of biological and genetic material.	**Cybersecurity Program**<br><br>**Laboratory Standard Operating Procedures**<br><br>**Employee Training**	No

CP & CCP Master Sheet

Control Point (CP) or Critical Control Point (CCP)	Cyberbiosecurity Critical Limits	Established Monitoring	Corrective Actions and Preventive Measures	HACCP Records	HACCP Verification
**CP-1**: Supplier Approval Program (control point)	Established verification program for received biological and genetic materials from known and verified sources.	All new suppliers must be verified prior to approval. Annual re-approval of all existing suppliers.	Appropriate processes at supplier to assure biological and genetic materials are maintained to assure cyberbiosecurity.	Supplier review and approval documents.	Supplier approval verification prior to receiving materials.
**CCP-1:** Alternative Supplier Verification of Biological and Genetic Materials Program	All alternative supply biological and genetic materials must be validated prior to use. With malware (data verification) and by genetic sequencing (material validation).	Every material shipment.	Materials or data not matching expected sequence review cannot be placed into use at the laboratory.	Alternative Supplier biological and genetic validation documentation.	Monthly review and signoff of alternative supplier validation documentation.
**CP-2:** Employee Training Program	Cybersecurity, cyber-physical security, biosecurity, and cyberbiosecurity training.	All new employees annually. All employees participate in quarterly threat assessment and review.	Untrained employees are not allowed into laboratory or to have access to biological and genetic material Untrained employees will also not have access to computer and software systems.	Training records on all employees.	Quarterly verification of training records on all employees.

**CP-3:** Security Programs	Cybersecurity, cyber-physical security, biosecurity, and cyber biosecurity programs.	On-going.	Best practices security programs. Programs reviewed and updated quarterly based on threat assessment.	Program verification, access records, antivirus software verification, threat assessment documentation.	Quarterly verification of Security Program records.
**CP-4**: Good Laboratory Standard Operating Procedures	Established and defended Laboratory Standard Operational Program	Daily review of laboratory notebooks and procedure records.	Procedure documentation by laboratory good practices with each analysis including handling of biological and genetic material.	Laboratory notebooks and SOP documentation.	Weekly review and verification of laboratory notebooks and laboratory procedures.
**CCP-2**: Cyberbiosecurity Data Verification Program	Verification of all external data prior to use or placement into internal data software systems.	Every data set.	Antivirus malware software should be used for all data received form external database and software systems.	Daily data verification documentation.	Annual reassessment of antivirus and on-going antivirus program updates.
